# RUTI Vaccination Enhances Inhibition of Mycobacterial Growth *ex vivo* and Induces a Shift of Monocyte Phenotype in Mice

**DOI:** 10.3389/fimmu.2019.00894

**Published:** 2019-04-30

**Authors:** Satria A. Prabowo, Hannah Painter, Andrea Zelmer, Steven G. Smith, Karin Seifert, Merce Amat, Pere-Joan Cardona, Helen A. Fletcher

**Affiliations:** ^1^Department of Immunology and Infection, Faculty of Infectious and Tropical Diseases, London School of Hygiene and Tropical Medicine, London, United Kingdom; ^2^Tuberculosis Centre, London School of Hygiene and Tropical Medicine, London, United Kingdom; ^3^Archivel Farma S.L., Badalona, Spain; ^4^Experimental Tuberculosis Unit (UTE), Fundació Institut Germans Trias i Pujol (IGTP), Universitat Autònoma de Barcelona (UAB), Badalona, Spain; ^5^Centro de Investigación Biomédica en Red (CIBER) de Enfermedades Respiratorias, Madrid, Spain

**Keywords:** RUTI, vaccine, tuberculosis, monocytes, mycobacteria, growth inhibition assay

## Abstract

Tuberculosis (TB) is a major global health problem and there is a dire need for an improved treatment. A strategy to combine vaccination with drug treatment, termed therapeutic vaccination, is expected to provide benefit in shortening treatment duration and augmenting treatment success rate. RUTI candidate vaccine has been specifically developed as a therapeutic vaccine for TB. The vaccine is shown to reduce bacillary load when administered after chemotherapy in murine and guinea pig models, and is also immunogenic when given to healthy adults and individuals with latent TB. In the absence of a validated correlate of vaccine-induced protection for TB vaccine testing, mycobacterial growth inhibition assay (MGIA) has been developed as a comprehensive tool to evaluate vaccine potency *ex vivo*. In this study, we investigated the potential of RUTI vaccine to control mycobacterial growth *ex vivo* and demonstrated the capacity of MGIA to aid the identification of essential immune mechanism. We found an association between the peak response of vaccine-induced growth inhibition and a shift in monocyte phenotype following RUTI vaccination in healthy mice. The vaccination significantly increased the frequency of non-classical Ly6C^−^ monocytes in the spleen after two doses of RUTI. Furthermore, mRNA expressions of Ly6C^−^-related transcripts (Nr4a1, Itgax, Pparg, Bcl2) were upregulated at the peak vaccine response. This is the first time the impact of RUTI has been assessed on monocyte phenotype. Given that non-classical Ly6C^−^ monocytes are considered to play an anti-inflammatory role, our findings in conjunction with previous studies have demonstrated that RUTI could induce a balanced immune response, promoting an effective cell-mediated response whilst at the same time limiting excessive inflammation. On the other hand, the impact of RUTI on non-classical monocytes could also reflect its impact on trained innate immunity which warrants further investigation. In summary, we have demonstrated a novel mechanism of action of the RUTI vaccine, which suggests the importance of a balanced M1/M2 monocyte function in controlling mycobacterial infection. The MGIA could be used as a screening tool for therapeutic TB vaccine candidates and may aid the development of therapeutic vaccination regimens for TB in the near future.

## Introduction

Tuberculosis (TB) remains a leading cause of death from infectious disease and is responsible for an estimated annual 1.6 million deaths globally (1). With the emergence of drug-resistant TB, there is a dire need for new therapy and for shorter, more effective, safer, and better tolerated treatment regimens. Therapeutic vaccination, which is a strategy to combine vaccination with drug treatment, could help to achieve these objectives and improve current treatments (2). TB vaccines are regarded to be equally effective against drug-sensitive and drug-resistant strains, due to the nature of drug-resistant mutations which are not considered to change the immunological profile of the organism (3). Therapeutic vaccination was first introduced by Robert Koch in his initial attempts to administer tuberculin to TB patients (4), and currently several therapeutic TB vaccine candidates are available in the vaccine pipeline.

The RUTI vaccine is among the few candidates currently on the clinical pipeline which is specifically developed as a therapeutic TB vaccine. The vaccine is composed of purified and liposomal cellular fragments of *Mycobacterium tuberculosis* (*Mtb*) bacilli cultured under stress (to mimic intra-granulomatous conditions) to induce latency antigens which would typically be hidden from the immune system (5). The immune response to RUTI has been studied in animal models and clinical studies and is characterized by a poly-antigenic response. Its main immunotherapeutic effect is thought to be through induction of a T helper-1 (Th1) response, not only against growth-related antigens but also structural antigens as shown in the murine model (2, 6). The prophylactic capacity of RUTI vaccine has also been evaluated in a murine model and the vaccination significantly reduced bacterial counts in both lungs and spleens following challenge with virulent bacilli (7). RUTI vaccination generated a poly-antigenic response in healthy human volunteers (phase I study), as well as in HIV-positive and HIV-negative patients with latent TB after isoniazid treatment for 1 month in a phase II clinical trial (8, 9).

The lack of an immune correlate of protection has been hampering the development of novel TB vaccines, as lengthy and expensive clinical trials with protracted follow-up periods are needed to demonstrate efficacy and proceed to licensure (10). For a single vaccine candidate, it generally takes at least a decade to reach efficacy trials from discovery (3, 10). If we are going to achieve the WHO target to eliminate TB by 2050, major progress is required to overcome the painstakingly slow progress. Such an immune correlate could also be used to help identify vaccine candidates with the greatest potential efficacy.

Mycobacterial growth inhibition assay (MGIA) has been developed as a simple and comprehensive tool to evaluate vaccine immunogenicity *ex vivo* (11, 12). As an assay that measures the summative vaccine-mediated host capacity to control mycobacterial growth, the MGIA is proposed as a screening tool for TB vaccine candidates (13–15). The nature of the *ex vivo* assay does not require that the vaccine-mediated immune mechanism which underlies growth control to be known in advance, while in turn the MGIA could help to determine immune mechanisms of protection through investigation of the cellular frequencies, phenotypes and cytokines that associate with enhanced growth inhibition (16–18). Several variations of human and murine MGIAs have been described in the literature [reviewed in (12)]. Here, we implemented the assay using direct co-culture of mouse splenocytes with mycobacteria, based on a recent optimization work (15), to investigate the potential of the RUTI vaccine to control mycobacterial growth *ex vivo*.

Monocytes are highly plastic and heterogeneous circulating cells, which are known to change their functional phenotype in response to environmental stimulation (19, 20). Two distinct subpopulations of mouse monocytes have been identified, commonly referred to as Ly6C^+^ and Ly6C^−^ monocytes (21). Ly6C^+^ monocytes represent classical pro-inflammatory and phagocytic monocytes which could subsequently differentiate into M1 macrophages, while Ly6C^−^ monocytes are regarded as non-classical anti-inflammatory monocytes which could differentiate into M2 macrophages (19). In addition to the induction of an antigen-specific Th1 response, evidence suggests the potential importance of a balanced M1/M2 monocyte function in controlling mycobacterial infection (20, 22). In a previous murine study, the RUTI vaccine was shown to reduce intragranulomatous infiltration and decrease Tumor Necrosis Factor (TNF)-α expression in *Mtb* infected mice (6). We hypothesize that immunization with RUTI would lead to improved control of mycobacterial growth *ex vivo* and such observation could be used to gain insight into the mechanism of immune protection.

In this study, we investigated the impact of RUTI vaccination in mice using the *ex vivo* MGIA assay and found an association between peak response of vaccine-induced growth inhibition and a shift in monocyte phenotype. Our study demonstrates the benefit of the *ex vivo* MGIA to aid the identification of immune mechanisms of action for therapeutic TB vaccine candidates. The MGIA could be used as a tool for screening such vaccine candidates and might aid the development of therapeutic vaccine regimens for TB patients.

## Materials and Methods

### Animals

Six to seven week-old female C57Bl/6 mice (Charles River, UK) acclimatized for at least 5 days were housed and handled in the Biological Services Facility (BSF) at London School of Hygiene and Tropical Medicine (LSHTM), UK. Mice were provided standard sterilized food and water *ad libitum*. Animals were housed in specific pathogen-free individually vented cages with environmental enrichment, with equal day and light cycle, at temperature between 19°-23°C and relative humidity of 45–65%. Mice were allocated to cages as groups of six. All animal work was carried out in accordance with the Animals (Scientific Procedures) Act 1986 under a license granted by the UK Home Office (PPL 70/8043), and approved locally by the LSHTM Animal Welfare and Ethics Review Body.

### Immunization

**Figure 1:**
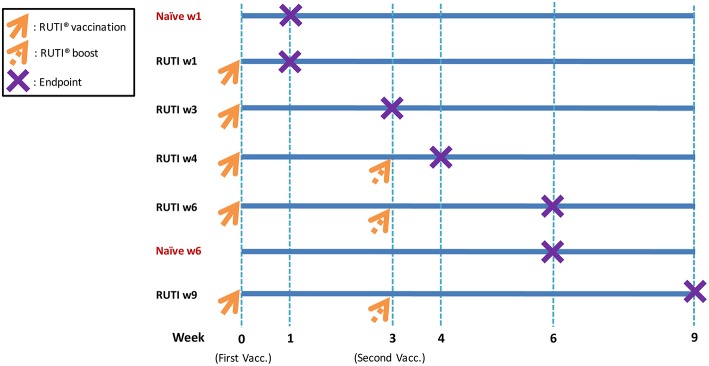
Experimental design and vaccination schedule. As indicated in the figure, orange arrows mean RUTI vaccination (dotted if boosting). The purple X represents endpoint (mice sacrifice). Enzyme-linked immunospot assay and mycobacterial growth inhibition assay were performed at each endpoint. In total, 42 mice were sacrificed at all time points (6 mice per group).

Seven experimental groups were established, with six mice per group (Figure 1). Mice in the treatment groups were vaccinated with RUTI, which is based on purified fragments of *Mtb* cultured under stress conditions and liposomed, manufactured by Archivel Farma (Badalona, Catalonia, Spain). Vaccination with RUTI (batch A14, 204 μg) was performed subcutaneously once or twice (3 weeks apart), as has been performed previously (6, 7). Five groups of mice were vaccinated with RUTI at week 0, among which three groups were boosted at week 3. Mice were sacrificed at week 1, 3, 4, 6, and 9 as the designated time points of this experiment. Two groups of mice sacrificed at week 1 and 6 served as naïve controls.

### Mycobacteria and Culture Conditions

Bacillus Calmette-Guérin (BCG) Pasteur strain was obtained from Aeras (Rockville, MD, USA) as frozen aliquots. These were stored at −80°C until needed. Mycobacterial suspensions for infection inoculum and BACTEC MGIT standards were prepared in antibiotic-free media (described below). All work with cells pre-BCG infection and involving BCG infected samples were performed in Biosafety Level (BSL) 2 laboratory.

### *Ex vivo* Mycobacterial Growth Inhibition Assay (MGIA)

At the determined time points, spleens were removed aseptically from mice and single splenocyte suspensions were prepared by homogenization through 100 μm cell strainers followed by lysis of red blood cells and washing. Cells were adjusted to 5 × 10^6^ splenocytes per 300 μl in antibiotic-free media [RPMI-1640 (Sigma-Aldrich, Dorset, UK) + 10% heat-inactivated FBS (Labtech International Ltd, Uckfield, UK) + 2 mM L-Glutamine (Fisher Scientific, Loughborough, UK)]. Mycobacteria were diluted in sufficient volume for all samples in the same media to a concentration of 90 CFU per 300 μl. Three hundred microliters aliquots of bacteria were added to the splenocytes, and the splenocytes-mycobacteria co-culture (600 μl) was then incubated in 48-well plates (Sigma-Aldrich, UK) at 37°C for 4 days.

After 4 days, splenocytes-mycobacteria mixtures were collected from the 48-well plates by pipetting up and down three times before transferring to 2 ml screw cap tubes. The tubes were centrifuged at 12,000 rpm in a bench top micro centrifuge and the supernatants were removed (500 μl) while ensuring the pellets remain intact. Five hundred microliters sterile tissue culture grade water was added to the 48-well plates which were incubated at room temperature for 5 min, followed by pipetting up and down for five times before transferring to the 2 ml screw cap tubes with pellets. The pellets were dissolved by pipetting and lysates containing mycobacteria were transferred to Bactec MGIT tubes supplemented with PANTA antibiotic and oleic acid-albumin-dextrose-catalase (OADC) enrichment broth [all from Becton Dickinson (BD), Oxford, UK]. The MGIT tubes were incubated in a Bactec MGIT liquid culture system (BD) until registered positive. The resulting time to positivity (TTP) was converted to bacterial numbers (log_10_ CFU) using a standard curve. The standard curve was obtained by a linear regression analysis of TTP values from inoculated BCG in 10-fold dilutions against CFUs obtained from plating aliquots of BCG onto 7H11 agar plates containing 10% OADC supplement (Yorlab, York, UK) and 0.5% glycerol. Direct-to-MGIT controls were included at each time point, defined as 90 CFU BCG directly placed into Bactec MGIT system without any pre-incubation (at day 0). To compare the growth inhibition between time points, log_10_ CFU values were normalized using the direct-to-MGIT controls by subtracting or adding the values based on the average TTP of direct-to-MGIT controls.

### Interferon (IFN)-γ ELISpot

To measure antigen-specific response toward mycobacterial antigen following RUTI vaccination over the time course, IFN-γ ELISpot assay was performed. Single cell suspensions of mouse splenocytes were resuspended in RPMI-1640 media containing 10% heat-inactivated FBS and 2 mM L-Glutamine. 96-well microtiter ELISpot plates (MAIPS4510, Millipore, Watford, UK) were coated with 10 μg/ml rat anti-mouse IFN-γ (clone AN18, Mabtech, Nacka Strand, Sweden). Free binding sites were blocked with the above mentioned media. 2.5 × 10^5^ of total splenocytes were added and incubated in duplicate with 10 μg/ml Purified protein derivative (PPD) (Oxford Biosystem, Oxfordshire, UK), RPMI media as a negative control, or phytohemaglutinin (PHA) (1 μg/ml, Sigma-Aldrich) and phorbol myristate acetate (PMA) (0.1 μg/ml, Sigma-Aldrich) as a positive control. Cells were incubated overnight at 37°C with 5% CO_2_. IFN-γ was detected with 1 μg/ml biotin labeled rat anti-mouse antibody (clone R4-6A2, Mabtech) and 1 μg/ml alkaline phosphatase-conjugated streptavidin (Mabtech). The enzyme reaction was developed with BCIP/NBT substrate (5-Bromo-4-chloro-3-indolyl phosphate/Nitro blue tetrazolium) (MP Biochemicals, UK) and stopped by washing the plates with tap water when individual spots could be visually detected (up to 3 min). Upon completion of the color development stage, spots were quantified using an automated plate reader with ELISpot 5.0 software. IFN-γ-specific cells are expressed as number of spot forming cells (SFC) per million splenocytes after non-specific background was subtracted using negative control wells.

### Flow Cytometry

Single splenocyte suspensions were fixed and red blood cells were lysed using PhosFlow lyse-fix solution (Becton Dickinson, Oxford, UK) for 30 min at 4°C prior to freezing. Fixed cells were then re-suspended in freezing media (FBS containing 10% DMSO) at the concentration of 10^6^ cells per ml and stored in a −40°C freezer from each time point. Frozen cells were thawed by adding FACS buffer (PBS containing 5% FBS) and pipetting up and down to encourage thawing. Cells were added to 10 ml of FACS buffer and centrifuged for 10 min at 1,800 rpm. Cells were re-suspended in FACS buffer (concentration 10^7^ cells/ml) and were left for 15 min on ice for rehydration.

Fc block (anti-mouse CD16/32, eBioscience, Loughborough, UK) was added to cells and left for further 10 min on ice prior to surface staining. Cells were aliquoted in FACS tubes (100 μl each, 10^6^ cells) and stained with the following titrated antibody: 1.25 μl CD3-APC/Cy7 (clone 17A2), 2.5 μl CD45R/B220-BV510 (clone RA3-6B2), 1.25 μl CD11b-PerCP/Cy5.5 (clone M1/70), 2.5 μl Ly6G-BV711 (clone 1A8), and 5 μl Ly6C-BV421 (clone HK1.4). All antibodies were purchased from Biolegend (via Fisher Scientific).

Cells were incubated for 30 min at RT in the dark and washed prior to analysis. Fluorescence minus one (FMO) controls were set using cells for each antibody and used to guide gating. OneComp beads (eBioscience, Loughborough, UK) were used to calculate compensation by staining with single antibodies as per manufacturer's instruction. Cells were acquired on a BD LSR II flow cytometer. Data was analyzed with FlowJo software version 10.4 (Treestar Inc., USA).

### Real-Time Quantitative PCR

To quantitatively analyse the mRNA expressions in splenocytes following RUTI vaccination, real-time quantitative reverse transcriptase PCR (qRT-PCR) assays were performed. 5 × 10^5^ splenocytes were stimulated overnight with PPD (final concentration 10 μg/ml). Cells were pelleted, lysed in 200 μl RLT buffer containing 10 μl/ml β-mercaptoethanol and stored in −40°C freezer from each time point. Cells were thawed and RNA was extracted using the RNAeasy mini kit (Qiagen, Manchester, UK) according to the manufacturer's instructions. After a DNAse treatment with RNase-free DNAse set (Qiagen, UK), total RNA concentration was determined by spectrophotometry with a Nanodrop (Labtech International, Heathfield, UK). One microgram of each sample of total RNA was reverse-transcribed into complementary DNA (cDNA) using Omniscript® Reverse Transcription kit (Qiagen, UK) according to the manufacturer's recommendation, using oligo(dT) (Invitrogen, UK) to obtain cDNA. Each PCR was carried out in a 20 μl volume in the presence of 10 μl of 2x QuantiTect SYBR Green PCR Master Mix (Qiagen, UK), 1 μl of cDNA (or water as a negative control), MgCl_2_ to a final concentration of 2.5 mM and primers to a final concentration of 0.5 μM. PCR was carried out for 10 min at 95°C denaturation, followed by 40 cycles at 95°C for 15 s, and 60°C for 1 min in Applied Biosystems 7500 (Applied Biosystems, CA, USA).

Analyses were performed for gene expressions of Nr4a1, Cebpb, Itgax, Pparg, Bcl2 (markers of Ly6C^−^), Ccr2, Sell, Ly6C2 (markers of Ly6C^+^), and β-actin (housekeeping gene). Primers used were listed in Table S1. mRNA expression of β-actin was quantified for every target sample to normalize for efficiency in cDNA synthesis and RNA loading. A ratio based on the β-actin mRNA expression was obtained for each sample.

### Statistical Analysis

Statistical analysis was carried out using GraphPad Prism version 7 (GraphPad, La Jolla, CA, USA). A *p*-value of < 0.05 was considered statistically significant. The specific tests used for each analysis are described in the figure legends.

## Results

### RUTI Vaccination Did Not Induce Antigen-Specific IFN-γ but Did Improve Mycobacterial Growth Inhibition in Murine Splenocytes

**Figure 2:**
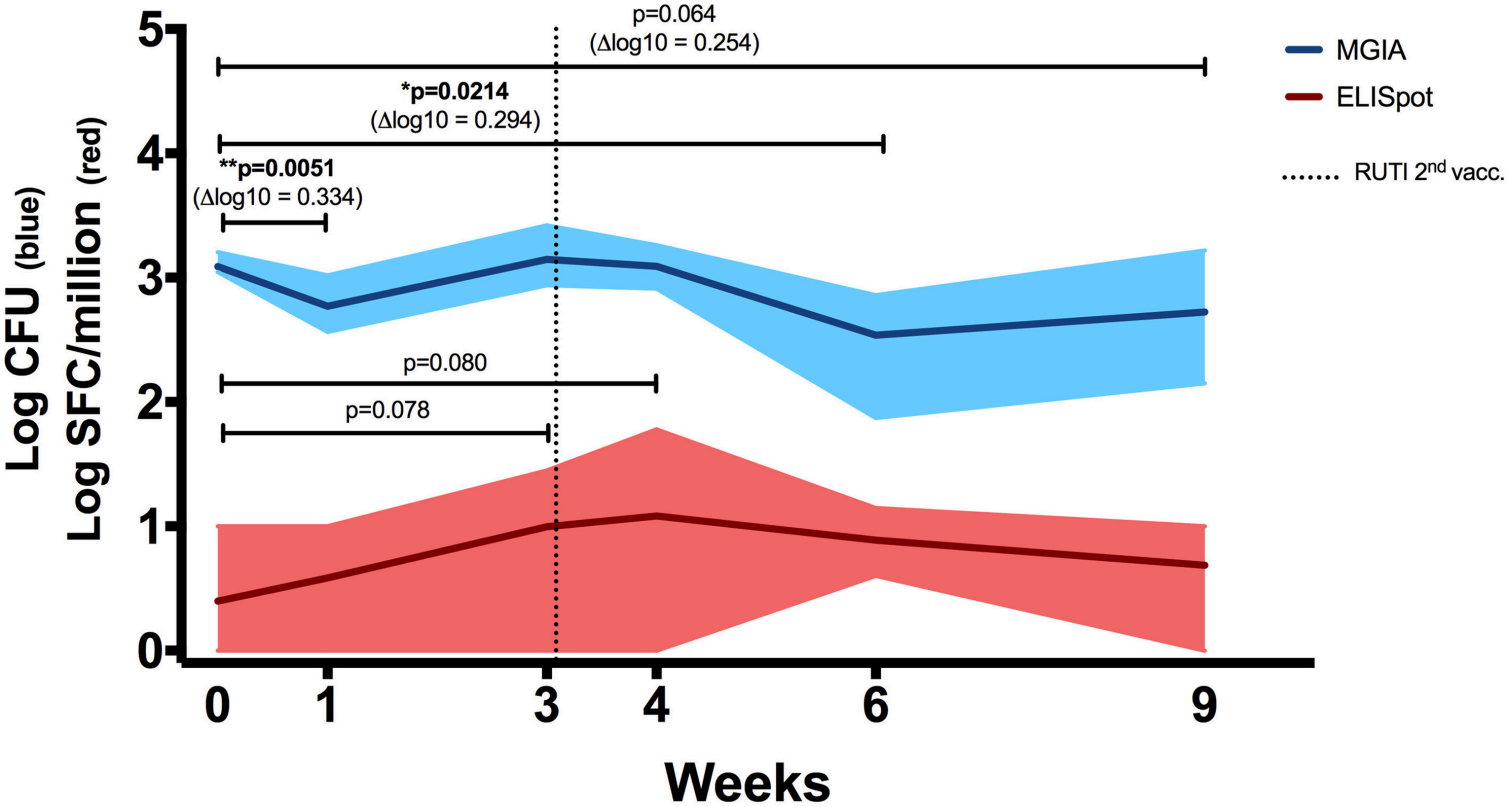
ELISpot (Red Line). IFN-γ response in mice receiving vaccination with RUTI was measured. Modest PPD antigen-specific responses were detected in splenocytes of healthy C57BL/6 mice across time points. The splenocytes were stimulated overnight with PPD, and the responses were detected using the IFN-γ ELISpot assay. SFC, spot-forming cells. Dark red line indicates mean response, and shading indicates range. Statistical significance was tested using Mann-Whitney test. MGIA (Blue Line**)**. RUTI vaccination induced mycobacterial growth inhibition in murine splenocytes, performed *ex vivo* in a 48-well plate. Dark blue line indicates mean mycobacterial growth, and shading indicates range. Time point 0 represents unvaccinated naïve-control mice sacrificed at week 1. One-way ANOVA was used to test for significance, followed by *t*-test. ^*^p < 0.05; ^**^p < 0.01.

To assess the immune response to mycobacterial antigens from mice vaccinated with RUTI, splenocytes were stimulated with PPD and the number of IFN-γ-producing cells was measured using the ELISpot assay (Figure 2, red line). We found a weak, non-significant response at 1 week following the second vaccination with RUTI (week 4, *p* = 0.08). This response appeared to have decreased by week 6. Significant control of mycobacterial growth was observed 1 week after the first vaccination and 3 weeks after the second RUTI vaccination (week 6) when compared to the baseline control (*p* = 0.0214, Figure 2, blue line). A trend of reduction was still observed 6 weeks after the second vaccination (week 9), although it did not reach significance (*p* = 0.064).

In a separate experiment performed in rotating tubes instead of 48-well plates (Figure S1A in Supplementary Material), RUTI-induced control of mycobacterial growth was superior to BCG-induced control when both vaccines were given 6 weeks prior to sacrifice (*p* < 0.005, Figure S1B). Therefore, week 6 appeared to be the peak response of RUTI in the *ex vivo* MGIA system. Growth control was also significant when compared to an age-matched control group (*p* < 0.05, Figure S2A in Supplementary Material).

RUTI vaccination led to control of mycobacterial growth as measured by the MGIA, despite a weak, non-significant antigen-specific IFN-γ response. This was consistent with results of a separate experiment with RUTI and BCG, in which BCG induced IFN-γ-secreting cells whereas RUTI did not and yet cells from RUTI immunized mice were better able to control mycobacterial growth than cells from BCG vaccinated mice (Figures S1C,D in Supplementary Material). The lack of robust IFN-γ response following two doses of RUTI in healthy mice has been observed previously during potency testing for batch release (Archivel Farma, personal communication). Then, four doses of RUTI were required for induction of a detectable IFN-γ response. Although IFN-γ is associated with control of TB infection in mice and reduces the risk of TB disease in humans (23–25), some studies suggested IFN-γ alone was not sufficient (26, 27). As the MGIA measures the summative effect of immune responses from all cellular components, our data implied an alternative mechanism by which growth inhibition could be enhanced *ex vivo* following RUTI vaccination.

### Shift of Monocyte Phenotype Following RUTI Vaccination in Healthy Mouse Splenocytes

**Figure 3:**
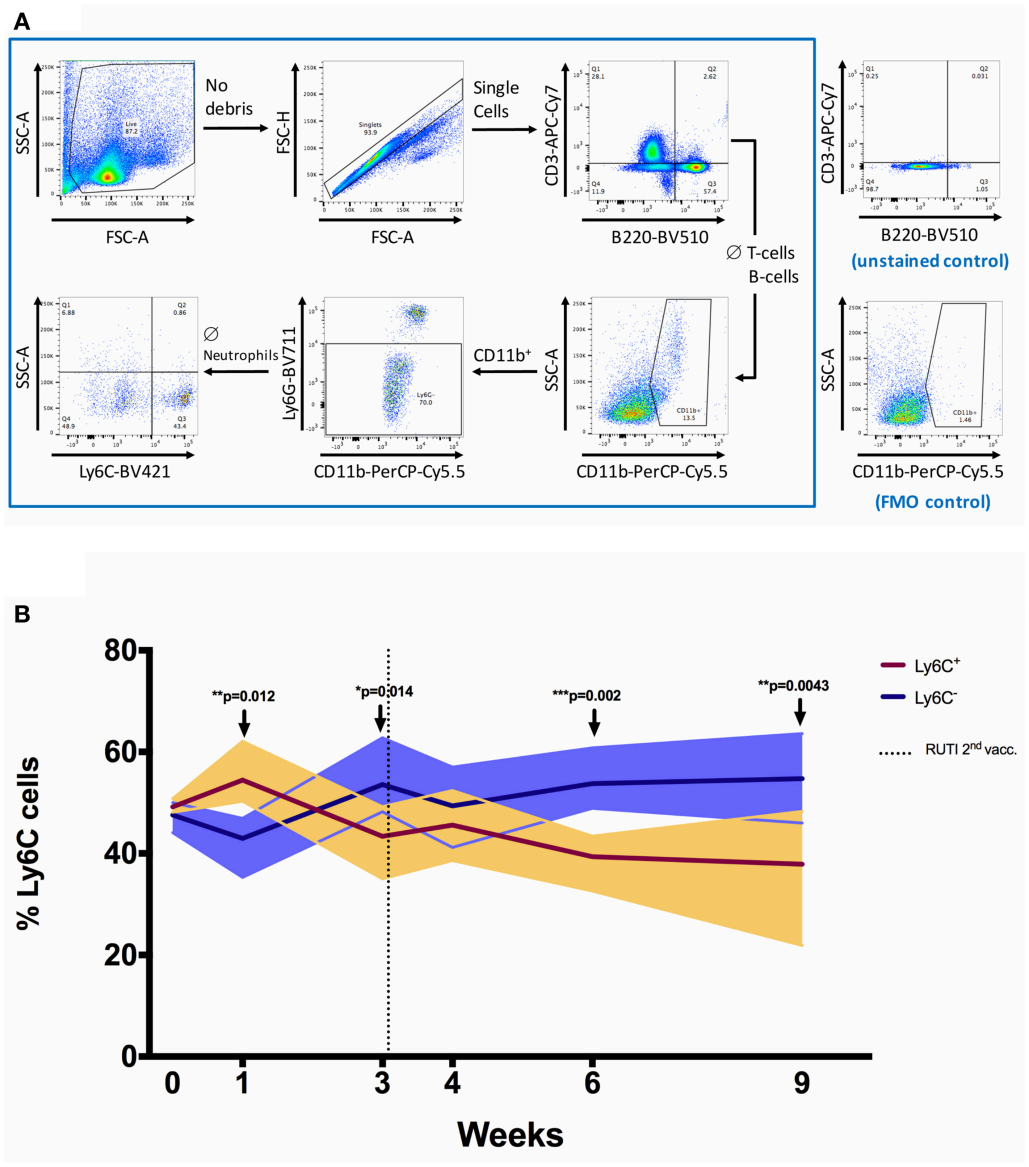
The shift of Ly6C^+^ and Ly6C^−^ monocytes/macrophages populations following RUTI vaccination in healthy mice. **(A)** Gating strategy for flow cytometric analysis. Splenocytes from C57BL/6 mice were fixed, stained and data acquired as described in Materials and Methods. Cell debris was gated out by use of FSC-SSC gate, followed by gating on single cells (FSC-A and FSC-H). A sequential gating strategy was then applied to determine the frequency of T-cells (CD3^+^), B cells (B220^+^), monocytes/macrophages (CD11b^+^ Ly6G^−^ ssc^low^), and the phenotypes of the monocytes/macrophages (Ly6C^+^ or Ly6C^−^) as a percentage of live cells. Plots shown are from a sample of a C57BL/6 spleen. **(B)** The frequencies of Ly6C^+^ and Ly6C^−^ monocytes/macrophages were compared at each time point following RUTI vaccination. Dark brown and dark purple lines represent mean percentages of Ly6C^+^ and Ly6C^−^ monocytes/macrophages, respectively and shading indicates range. Time point 0 represents unvaccinated naïve-control mice sacrificed at week 1. Statistical significance was tested using unpaired *t*-test, ^*^p < 0.05; ^**^p < 0.01; ^***^p < 0.005.

In this experiment, we investigated the impact of RUTI vaccination on the population of immune cells in the spleen using flow cytometry (Figure 3 and Figure S3 in Supplementary Material). RUTI did not appear to alter the percentages of monocytes/macrophages, T-cells, and B-cells in the spleen of healthy mice (*p* > 0.05, Figures S3A,C,D). We also measured monocyte to lymphocyte (ML) ratio as a factor influencing mycobacterial growth inhibition (28, 29), defined as the percentage of monocytes/macrophages divided by the percentage of T-cells and B-cells. We did not find a significant change of ML ratio following RUTI vaccination across time (Figure S3B).

We then further characterized monocyte phenotype based on the Ly6C marker, and observed a significant increase of Ly6C^−^ monocytes/macrophages (non-classical) at weeks 3, 6, and 9 following RUTI vaccination (*p* < 0.05, Figure 3B). The peak increase of Ly6C^−^ cells was observed at week 6, with the shift being evident compared to both baseline and age-matched naïve control at week 6 (Figure S2B in Supplementary Material). The Ly6C^+^ monocytes/macrophages (classical) population appeared to be decreasing following vaccination, although there was an initial significant increase observed at week 1 (*p* < 0.05, Figure 3). The shift of Ly6C^+^/Ly6C^−^ phenotype at week 6 was notably consistent with the peak response of the *ex vivo* mycobacterial growth inhibition assay, in which enhanced inhibition was observed following two doses of RUTI vaccination. We found a non-significant correlation between higher frequency of Ly6C^−^ monocytes/macrophages and lower growth of mycobacteria across time points (*p* = 0.247, Spearman *r* = −0.20, data not shown).

### Gene Expression of Ly6C^+^- and Ly6C^−^- Related Markers Induced by RUTI Vaccination

To confirm findings from the flow cytometry analysis described in the previous section, we performed real-time qRT-PCR looking at transcripts associated with Ly6C^+^ and Ly6C^−^ monocytes/macrophages. The selection of transcripts was based on a recent publication by Mildner et al. (21) regarding genomic characterization of murine monocytes. We chose differentially expressed transcripts between the two monocyte subsets from several gene clusters of monocyte development. Three transcripts, namely Nr4a1, Itgax, and Pparg, were selected from a cluster which was strongly upregulated in Ly6C^−^ compared to Ly6C^+^ monocytes. Cebpb was selected from a cluster that showed a gradual increase of expression from Ly6C^+^ to Ly6C^−^ monocytes. Bcl2 belongs to a cluster characterized by transcripts associated with a progenitor phenotype of MDP (monocyte-macrophage DC progenitor), which was highly expressed in Ly6C^−^ monocytes. In addition, transcripts associated with Ly6C^+^ monocytes were selected from clusters involved in cell cycle (Sell and Ly6C2) as well as maturation of Ly6C^+^ monocytes (Ccr2).

**Figure 4:**
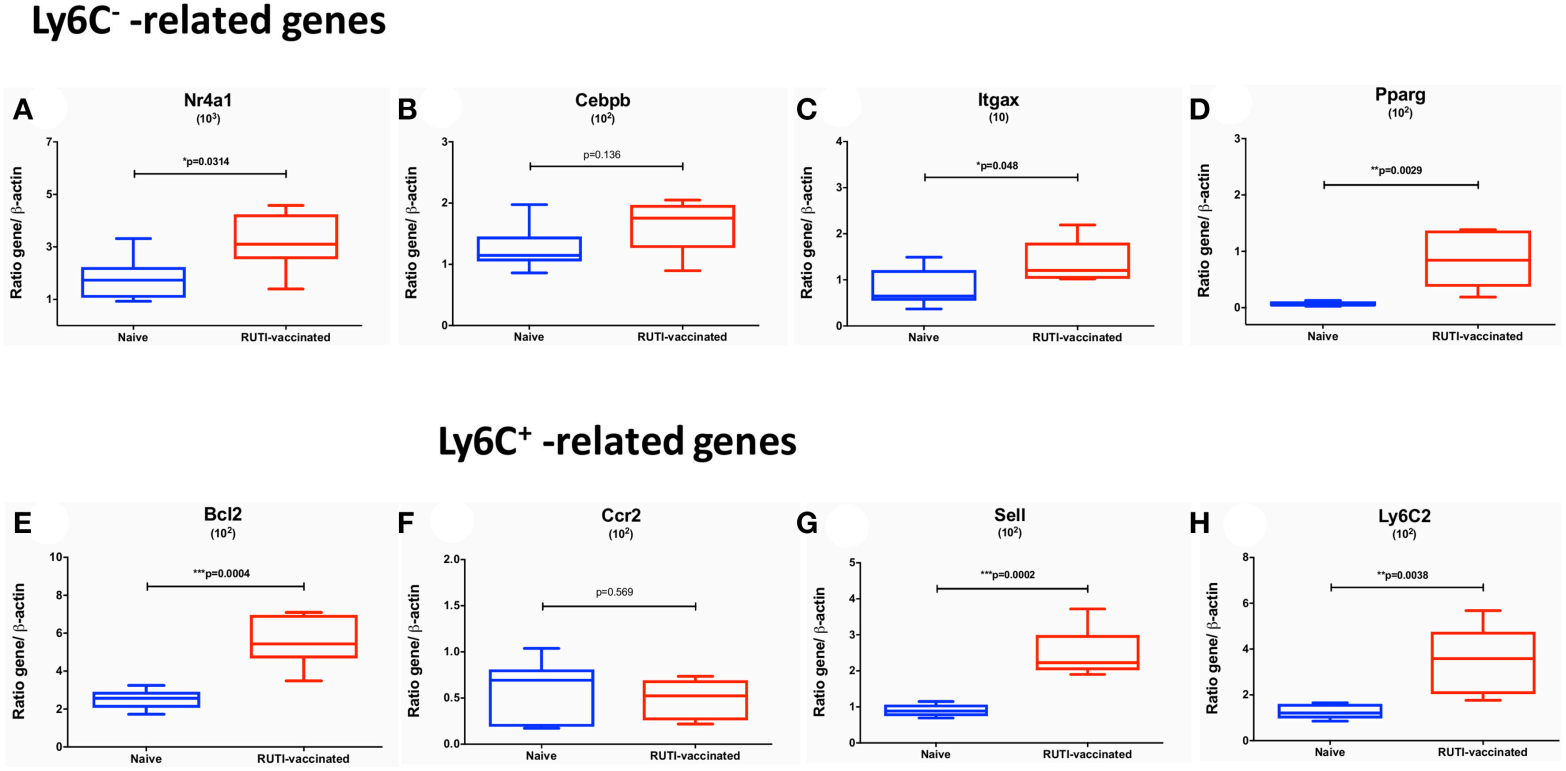
mRNA expressions of Ly6C^−^ -related **(A–E)** and Ly6C^+^ -related genes **(F–H)** in mice following vaccination with RUTI at week 6 compared to the age-matched naïve control group. Data are expressed as ratio obtained after dividing every value by the expression of β-actin in each sample and multiplying it by a factor (ranging from 10^1^ to 10^3^). The box plots show the minimum and maximum values (ends of the whiskers), the median (band near the middle of the box) and interquartile ranges. Statistical significance was tested using unpaired *t*-test, ^*^p < 0.05; ^**^p < 0.01; ^***^p < 0.005.

The mRNA expressions of Ly6C^−^-related transcripts, including Nr4a1, Itgax, Pparg, Bcl2, were significantly upregulated following the second RUTI vaccination at week 6 (*p* < 0.05, Figures 4A,C–E), with a trend of upregulation for Cebpb (*p* = 0.136, Figure 4B). While we did not observe a difference in expression of Ccr2, a Ly6C^+^ -related gene, following RUTI vaccination at the peak time point, we saw significant upregulations of Sell and Ly6C2 at week 6 (*p* < 0.05, Figures 4G–H).

## Discussion

Vaccination with RUTI resulted in enhanced control of mycobacterial growth, notably at 3 weeks following the second vaccination (week 6), which was considered as the peak response of the vaccine observed in the *ex vivo* assay system. In addition, there was also a significant reduction of mycobacterial growth at 1 week after the first vaccination. There was no significant induction of antigen-specific IFN-γ as measured by ELISpot or ELISA. These observations were replicated in a separate experiment and support the notion that the MGIA measures different aspects of immunity following vaccination as compared to the IFN-γ-based assay, with the MGIA being reflective of the summative effect of host immune responses at the point of tissue harvest, compared to the IFN-γ-based assay which is an assessment of a T-cell mediated recall response following vaccination.

In this context, we argue that the *ex vivo* assay is more capable of measuring the direct, short-term effect of vaccination, while the IFN-γ response is more representative of the medium- to long-term protection conferred by vaccination. This was supported by a recent finding of Fletcher et al. in which antigen-specific IFN-γ response was associated with reduced risk of developing TB disease in BCG-vaccinated South African infants (25). In testing therapeutic vaccine candidates, it might be more relevant to measure the direct effect of vaccination, as it would represent the immediate and potentially synergistic impact of a vaccine during TB chemotherapy. This was depicted in the *ex vivo* assay, in which significant mycobacterial growth inhibition was observed at 1 week following the first RUTI vaccination, when there was no apparent increase of IFN-γ response at this time point.

The *ex vivo* MGIA was shown to be able to capture the impact of distinct aspects of protective immunity as a part of a summative measurement of cellular immune responses. In this study, we used BGC as the immune target of the growth inhibition assay, as previous studies have shown a good concordance of growth between BCG and *Mtb* in the MGIA assay when both mycobacteria were used to assess responses following vaccination and treatment (30–32). In addition, studies by Marsay et al. and Zelmer et al. also showed that the *ex vivo* growth inhibition using BCG as immune target was correlated with *in vivo* growth in mouse challenge experiment using *Mtb* Erdman (15, 16). In our experiment, we performed flow cytometry to characterize immune cells associated with enhanced growth inhibition across time. RNA was also isolated to investigate gene expression by RT-qPCR. The important finding in our present study was the impact of RUTI vaccination in shifting the phenotype of Ly6C^+^/Ly6C^−^ monocytes/macrophages in the spleen of healthy mice. This was the first time the impact of RUTI on monocyte phenotype has been assessed. In addition, while others have shown a lack of correlation between antigen-specific IFN-γ and mycobacterial growth inhibition, this is the first demonstration of vaccine induced mycobacterial growth inhibition in the absence of antigen-specific IFN-γ. Our results suggest that the enhanced control of mycobacterial growth *ex vivo* was associated with the increase of Ly6C^−^ monocytes (non-classical, anti-inflammatory), which were both observed 3 weeks after the second vaccination with RUTI (week 6).

We confirmed the flow cytometry finding by demonstrating the upregulation of transcripts associated with Ly6C^−^ cells at the peak time point (week 6), while one of the Ly6C^+^ gene transcript (Ccr2) remained unchanged. Among the significantly upregulated transcripts in our study was Nr4a1, which is obligatory for Ly6C^−^ monocytes development (33). Expression of Nr4a1 as a monocyte survival factor is regulated by Cebpb (21, 34), which was also elevated following RUTI vaccination. In addition, the expressions of all other Ly6C^−^-associated transcripts (Itgax, Pparg, Bcl2) were significantly upregulated following vaccination in our study. As Ly6C^−^ monocytes are known to mature from Ly6C^+^ (35, 36), the increased expression of some Ly6C^+^ transcripts (Sell and Ly6C2) at the peak time point was regarded as a vestige of the transition from Ly6C^+^ to Ly6C^−^. Ly6C^−^ monocytes do not represent a distinct lineage and instead arise from the conversion of Ly6C^+^ cells (37) and approximately 92% of expressed transcripts are shared between the two monocyte subsets (38). Our results suggest that RUTI could enhance Ly6C^+^ cell frequency and induce maturation of Ly6C^+^ to Ly6C^−^ monocytes. This was evidenced by our results in which an initial increase of Ly6C^+^ monocytes was observed at week 1 following the first RUTI vaccination, followed by the shift toward Ly6C^−^ and the decrease of Ly6C^+^ monocytes at the subsequent time points.

Ly6C^−^ monocytes secrete anti-inflammatory cytokine upon bacterial infection *in vivo* and when recruited to tissue, are more likely to differentiate into M2 macrophages (35). This is in contrast to Ly6C^+^ monocytes, which are more likely to mature into pro-inflammatory M1 macrophages (36). In relation to our findings, the study by Guirado et al. (6) demonstrated a decrease of intragranulomatous infiltration in the lungs upon administration of RUTI in infected mice after treatment. One of the notable findings in that study was a significant decrease of TNF-α at the earliest time point after RUTI administration, measured by mRNA expression in the lung. Among the producers of TNF-α during bacterial infection are Ly6C^+^ monocytes which will subsequently differentiate into M1 macrophages (19, 39). Although our experiment differs with the one previously performed by Guirado and colleagues in several aspects (including the investigated target organ), the decrease of TNF-α observed in the previous study could be associated with the impact of RUTI on monocyte populations which has been discovered in our investigation.

RUTI is a poly-antigenic vaccine made from fragmented *Mtb* bacilli designed to induce host immune response against latency epitopes, which is grown in stress, purified and liposomed. The part of RUTI formulation that could potentially influence monocyte phenotype remains to be further explored. Nevertheless, our study has revealed an interesting mechanism of action of RUTI as a therapeutic vaccine for TB. As reviewed by Prabowo et al. (40), it is essential to prevent the occurrence of exacerbated immune response for a successful therapeutic vaccination strategy in TB. This was exemplified in a recent study, in which an excessive inflammation from the T-cell compartment could also be deleterious in TB (41). The fact that RUTI vaccination could induce a shift toward an anti-inflammatory monocyte phenotype might be considered as an advantage in this context and such approach should be further investigated in future studies.

While this could imply that less inflammation might be beneficial for the *ex vivo* control of mycobacterial growth following RUTI vaccination in healthy mice, our results should be interpreted with prudence in relation to previous results of RUTI testing in *in vivo* animal models. The observed trend of correlation between the frequency of Ly6C^−^ monocytes/macrophages and growth of mycobacteria across time points hinted that this was only one aspect contributing to the enhanced *ex vivo* growth control and other factors might be playing roles. In a murine model infected with *Mtb*, RUTI has been shown to trigger a balanced Th1/Th2 response as well as Immunoglobulin (Ig)G1, IgG2a and IgG3 antibodies against 13 *Mtb* antigens, reflecting its broad immunogenicity (42). In addition, the vaccine was also shown to induce a Th3 response as a subset population of regulatory T-cells (42). In another mouse study, RUTI administration following drug therapy in infected mice stimulated stronger IFN-γ secretion by CD4^+^ and CD8^+^ T-cells compared to BCG against early secretory antigen target (ESAT)-6, Ag85B, and PPD and also induced an immune response against structural antigens Ag16 kDa and Ag38 kDa (6). While immune responses in infected and drug-treated mice could be reasonably different in comparison to healthy mice as was done in our *ex vivo* study, the fact that RUTI did not induce an exacerbated immune response in various animal studies could be linked to its impact on Ly6C^−^ monocytes which was discovered in our investigation. In the experimental animals immunized with RUTI, no elevated IgE levels were observed (43). In this study, histology also revealed no eosinophilia, necrosis or granulomatous infiltration, as well as allergic or hypersensitivity reactions. Taken together, these findings and observations suggest that RUTI could induce a balanced immune response, promoting an effective cell-mediated response whilst at the same time limiting an excessive inflammation, which could be beneficial for its implementation as a therapeutic vaccine for active TB patients undergoing treatment.

Intriguingly, a recent study by Joosten et al. demonstrated the protective effect of human non-classical CD14^dim^ monocytes in inhibiting mycobacterial growth following recent *Mtb* exposure and BCG vaccination through the trained innate immunity mechanism (44). As Ly6C^−^ monocyte is the equivalent of CD14^dim^ monocyte in mouse (19), our results could also suggest an impact of RUTI on trained innate immunity which has not been characterized before. BCG is known to induce trained immunity on human monocytes and such ability has been attributed to the non-specific protective effect conferred by the vaccine (45, 46). A synthetic mycobacterial structure—muramyl tripeptide (MTP)—is also considered to induce trained immunity (47), and interestingly, both MTP and RUTI are delivered in liposome. Further studies are required to elucidate downstream and upstream pathways related to the potential effect of RUTI on trained innate immunity.

In summary, we have demonstrated the benefit of the *ex vivo* MGIA assay to help streamlining and identifying immune mechanisms of a therapeutic TB vaccine candidate. Our results could be complemented by further experiments, such as by using cells from infected mice. Depleting Ly6C^−^ monocytes and using genetically deficient mice could also provide more insight into the mechanism of protection by Ly6C^−^ monocytes in the *ex vivo* assay system. Future investigation of the novel immune mechanism of RUTI observed in our study is also warranted in upcoming clinical trials of the vaccine and could potentially accelerate the development of a therapeutic vaccine regimen for TB patients in the near horizon.

## Author Contributions

SP and HF conceived and planned the experiments. SP performed the experiments, with HP and AZ participating in some of the qPCR and MGIA experiments. SP, HF, SS, KS, MA, and P-JC contributed to the interpretation of the results. SP took the lead in writing the manuscript. All authors reviewed, provided critical feedback and approved the final version of the manuscript.

### Conflict of Interest Statement

MA is an employee of Archivel Farma S.L. P-JC is a consultant of Archivel Farma S.L. and a co-inventor of the patent of RUTI as a therapeutic vaccine. The remaining authors declare that the research was conducted in the absence of any commercial or financial relationships that could be construed as a potential conflict of interest.
