# Supplementary material for: RUTI Vaccination Enhances Inhibition of Mycobacterial Growth ex vivo and Induces a Shift of Monocyte Phenotype in Mice
Source: Front Immunol. 2019 Apr 30;10:894. doi: 10.3389/fimmu.2019.00894 (PMC6503078; doi:10.3389/fimmu.2019.00894)
Supplement: Supplementary file 1 [file Data_Sheet_1.docx]

**Supplementary Material**

**RUTI vaccination enhances inhibition of mycobacterial growth *ex vivo* and induces a shift of monocyte phenotype in mice**

Authors: Satria A. Prabowo^a,b*^, Hannah Painter^a,b^, Andrea Zelmer^a,b^, Steven G. Smith^a,b^, Karin Seifert^a,c^, Merce Amat^d^, Pere-Joan Cardona^e,f^, Helen A. Fletcher^a,b^

^a^ Department of Immunology and Infection, Faculty of Infectious and Tropical Diseases, London School of Hygiene and Tropical Medicine, London, UK

^b^ Tuberculosis Centre, London School of Hygiene and Tropical Medicine, London, UK

^c^ Current address: Federal Institute for Drugs and Medical Devices, Bonn, Germany

^d^ Archivel Farma S.L., Badalona, Catalonia, Spain

^e^ Experimental Tuberculosis Unit (UTE), Fundació Institut Germans Trias i Pujol (IGTP), Universitat Autònoma de Barcelona (UAB), Badalona, Catalonia, Spain

^f^ Centro de Investigación Biomédica en Red de Enfermedades Respiratorias (CIBERES), Madrid, Spain

# *corresponding author: [satria.prabowo@lshtm.ac.uk](mailto:satria.prabowo@lshtm.ac.uk)

**Tables**

**Table 1.** Specific sets of primers of real-time PCR. F, forward primer; R, reverse primer.

| **Gene names** | **Primer Sequences** |
| --- | --- |
| **Ly6C^-^ related** | |
| Nr4a1 | (F)GCACAGCTTGGGTGTTGATG (R)CAGACGTGACAGGCAGCTG |
| Cebpb | (F)GCTGAGCGACGAGTACAAGA (R)TGCTCCACCTTCTTCTGCAG |
| Itgax | (F)TTTGGGTGCCCATAGAGCTG (R)ATACCTGAGGGTGGGAGACC |
| Pparg | (F)TCTCTCCGTAATGGAAGACC (R)GCATTATGAGACATCCCCAC |
| Bcl2 | (F)AGGATTGTGGCCTTCTTTGA (R)CAGATGCCGGTTCAGGTACT |
| **Ly6C^+^ related** | |
| Ccr2 | (F)AGAGAGCTGCAGCAAAAAGG (R)GGAAAGAGGCAGTTGCAAAG |
| Sell | (F)TCAGACTCCTTGCGCATAG (R)GTGGCTGTCACTCACAGATAG |
| Ly6C2 | (F)TGCCTCGGTCTTCCAAGTTC (R)ACTTCTTATGCAGGGGCCAC |
| **Housekeeping gene** | |
| β-actin | (F)CATCCGTAAAGACCTCTATGCCAAC (R)ATGGAGCCACCGATCCACA |

**Figures**


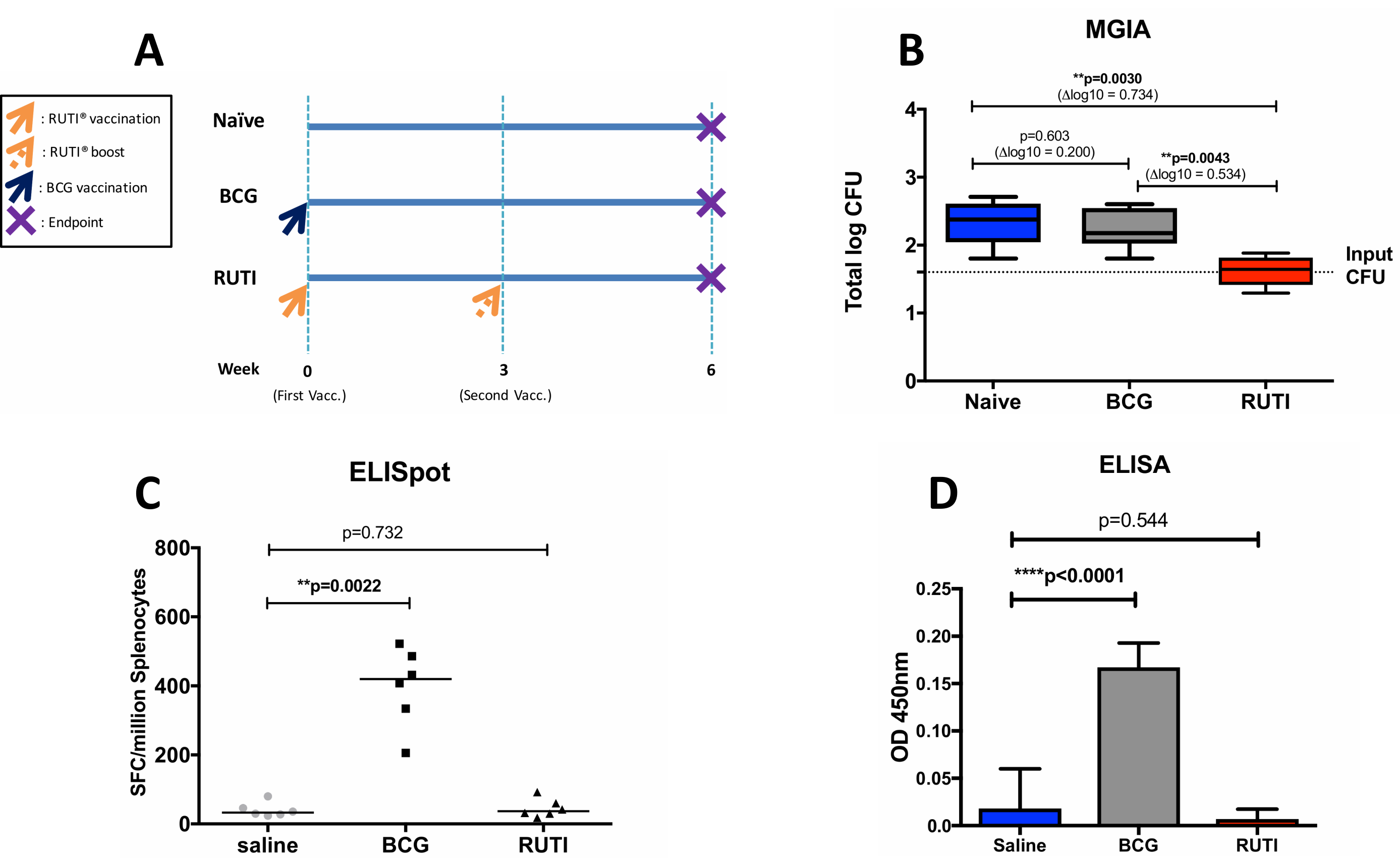


**Figure S1. RUTI vaccination induced mycobacterial growth inhibition in murine splenocytes in comparison with BCG, performed in rotating tubes.** Groups of mice were immunised with BCG, RUTI or placebo/ saline **(A)**. RUTI vaccination enhanced mycobacterial growth inhibition which was superior to BCG **(B)**. IFN-γ responses in mice receiving vaccination with RUTI and BCG were assessed using ELISpot and ELISA assays **(C and D)**. Statistical significance was tested using t-test (B and D) and Mann-Whitney test (C). A p value <0.05 was considered statistically significant.

**
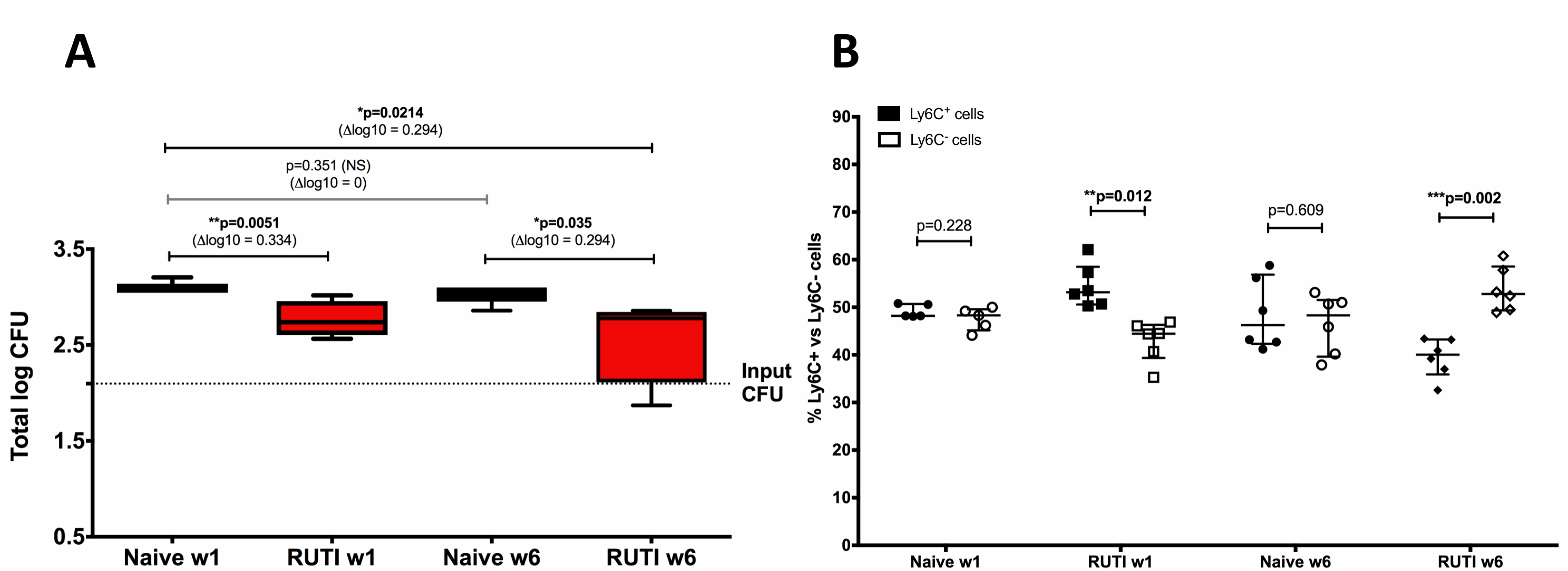
**

**Figure S2. (A)** An additional naïve group was set up at the peak time point (week 6). No difference in growth inhibition was observed between the naïve group at week 1 and week 6. The box plots show the minimum and maximum (ends of the whiskers), the median (band near the middle of the box) and interquartile ranges. **(B)** The proportion of Ly6C^+^ and Ly6C^-^ monocytes/macrophages was not shifted in the naïve groups on week 1 and week 6, in contrast to the RUTI-vaccinated groups. Error bars represent the median +/- interquartile range. Statistical significance was tested using t-test. A p value <0.05 was considered statistically significant. *p < 0.05; **p < 0.01; ***p < 0.005.

**
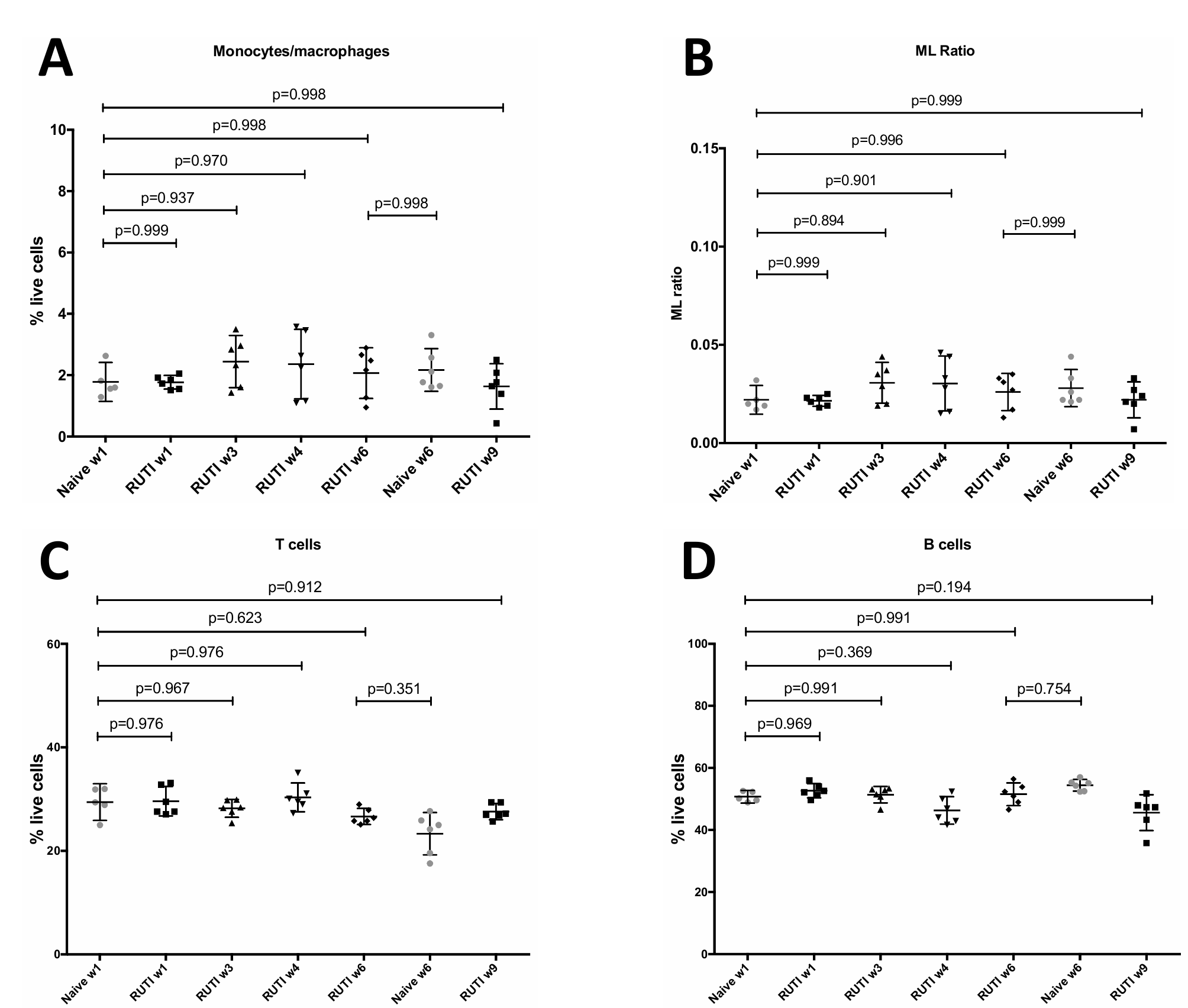
**

**Figure S3.** The frequency of monocytes/macrophages **(A)**, ML ratio **(B)** as well as the frequency of T cells **(C)** and B cells **(D)** were determined in spleen using the gating strategy described in Figure 3. The frequencies of monocytes/macrophages (CD11b^+^ Ly6G^-^ ssc^low^), T cells (CD3^+^) and B cells (B220^+^) were used to calculate the ML ratio. The ML ratio was obtained by dividing the percentage of monocytes/macrophages by the sum of the percentages of T and B cells. Error bars represent the median +/- interquartile range. p values were determined using ordinary ANOVA. A p value <0.05 was considered statistically significant.
